# What happens to all that propofol during prolonged sedation?

**DOI:** 10.1186/cc9778

**Published:** 2011-03-11

**Authors:** N Cowley, TH Clutton-Brock

**Affiliations:** 1 University Hospital Birmingham, UK

## Introduction

There are few published data on the pharmacokinetics of propofol infusion for prolonged periods in critical care. Propofol is frequently infused for days or weeks in critically ill patients with organ dysfunction. We aimed to determine whether propofol concentrations in critically ill patients are predictable during constant rate infusion, and whether significant organ failure might lead to accumulation when compared with conventional pharmacokinetic models.

## Methods

We compared blood propofol levels with total dose and duration of propofol infusion in 53 samples from 43 patients on a mixed critical care unit undergoing prolonged sedation. Estimated propofol concentration was calculated using the Marsh algorithm. The Richmond Agitation Scale at the point of propofol measurement was recorded, and the Sequential Organ Failure Assessment (SOFA) score was recorded for assessment of its impact on propofol levels.

## Results

**Figure 1:**
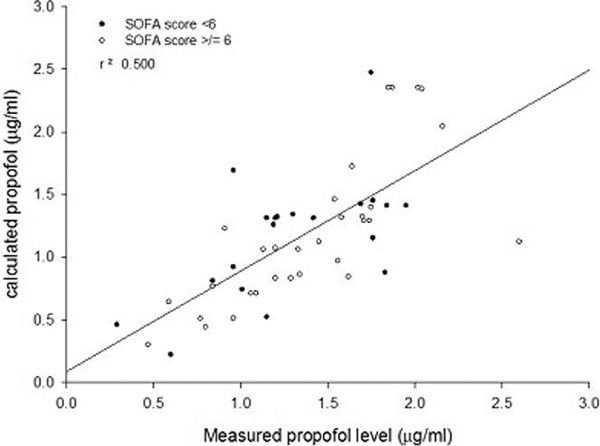
**Correlation between measured and estimated propofol levels in critically ill patients**.

Propofol was infused for a mean of 33 hours (14 to 44 interquartile range). The mean measured propofol concentration was 1.37 μg/ml (range 0.29 to 2.60). There was fairly good correlation between estimated propofol concentrations (based on the Marsh model) and measured levels with a *R*^2 ^value of 0.500, shown in Figure 1. The level of organ failure did not impact significantly on the accuracy of predicted propofol levels.

## Conclusions

We were able to demonstrate a correlation between predicted propofol levels and those measured in blood. Predicted propofol levels were on average lower than measured levels, suggesting a reduced capacity to metabolise propofol in critical illness, although this effect was not marked, and we were unable to demonstrate an association between severity of organ failure and deviation of measured from predicted propofol levels.
